# Glycated Hemoglobin and Cardiovascular Disease in Patients Without Diabetes

**DOI:** 10.3390/jcm14010053

**Published:** 2024-12-26

**Authors:** Grzegorz K. Jakubiak, Artur Chwalba, Aleksandra Basek, Grzegorz Cieślar, Natalia Pawlas

**Affiliations:** 1 Department of Pharmacology, Faculty of Medical Sciences in Zabrze, Medical University of Silesia, Jordana 38 St., 41-800 Zabrze, Poland; artur.adam.chwalba@gmail.com (A.C.); n-pawlas@wp.pl (N.P.); 2 Department of Internal Medicine, Angiology, and Physical Medicine, Faculty of Medical Sciences in Zabrze, Medical University of Silesia, Batorego 15 St., 41-902 Bytom, Poland; basek.aleksandra@interia.pl (A.B.); cieslar1@tlen.pl (G.C.)

**Keywords:** glycated hemoglobin, atherosclerotic cardiovascular disease, intima-media thickness, arterial stiffness, pulse wave velocity, ankle–brachial index, endothelial dysfunction

## Abstract

Cardiovascular diseases (CVDs) are one of the most critical public health problems in the contemporary world because they are the leading cause of morbidity and mortality. Diabetes mellitus (DM) is one of the most substantial risk factors for developing CVDs. Glycated hemoglobin is a product of the non-enzymatic glycation of hemoglobin present in erythrocytes. The determination of the percentage of glycated hemoglobin (HbA1c) is commonly used in clinical practice to assess glycemic control in patients diagnosed with DM. This method is much more informative than repeated blood glucose tests, because the HbA1c value reflects the degree of glycemic control over the last three months. It is, therefore, not surprising that the HbA1c value correlates with the presence and severity of diabetes complications, including CVDs, in the population of diabetic patients. The purpose of this publication was to present the results of a literature review on the relationship between the HbA1c value in people without DM, the presence and severity of subclinical cardiovascular dysfunction, and the presence of clinically overt CVDs. The most important tools used to assess subclinical cardiovascular dysfunction included the measurement of intima-media thickness (IMT), especially carotid IMT (cIMT), arterial stiffness assessment by the measurement of pulse wave velocity (PWV), and ankle–brachial index (ABI). According to the results of the studies cited in this literature review, it can be concluded that there are certain relationships between HbA1c, the presence and severity of subclinical cardiovascular dysfunction, and the presence of clinically overt CVDs such as coronary heart disease, cerebrovascular disease, and chronic lower extremity ischemia in non-diabetic patients. It is worth noting, however, that the results of studies conducted so far in this area are not fully unambiguous. Further studies are needed to better understand the influence of additional factors on the relationship between HbA1c and cardiovascular dysfunction in non-diabetic patients.

## 1. Introduction

Diabetes mellitus (DM) is a heterogeneous group of diseases for which the typical exponent is a set of metabolic disorders secondary to chronic hyperglycemia [1]. Although chronic hyperglycemia is the essence and diagnostic criterion of diabetes, diabetes is not only a disorder of the carbohydrate metabolism. The pathophysiology of diabetes consists of complex disorders of carbohydrates, lipids [2,3], proteins [4], and nucleic acids [5,6] metabolism, and, thus, all macromolecules in the human body.

Glycated hemoglobin is the product of the irreversible non-enzymatic glycation of hemoglobin. Glycated hemoglobin is a parameter measured in routine clinical practice that reflects the mean glycemia value during the three months preceding the determination, corresponding to the approximate lifetime of a single erythrocyte [7]. Therefore, the percentage of glycated hemoglobin (HbA1c) reflects the degree of glycemic control in a patient living with diabetes better than the point measurement of glycemia [8]. The HbA1c result is given in relative units (mmol/mol) or as a percentage (%) relative to the total hemoglobin concentration in the blood. Table 1 presents the relationship between the HbA1c value and the mean serum glucose concentration according to the results published in 2008 by Nathan et al. [9].

Chronic hyperglycemia leads to numerous chronic complications, the central parts of which are the consequences of the development of microangiopathy and macroangiopathy [10]. As a result of the development of atherosclerosis, ischemic heart disease (including acute coronary syndromes) and peripheral arterial disease may develop, which may manifest as cerebral circulation disorders, ischemia of the lower limbs, and intestinal and kidney ischemia [11]. In the course of DM, there is a significant acceleration of the development of atherosclerosis and the modification of its course. In the case of atherosclerosis of the arteries of the lower extremities in diabetic patients, the lesions are more often multi-level, more often severe stenoses occur below the knee, and calcifications of the middle layer of the arterial wall develop [12]. For patients who are treated with endovascular methods (angioplasty with optional stent implantation), DM is a significant risk factor for restenosis, which may lead to the need for re-intervention and limit the effectiveness of treatment [13].

The risk of developing complications of diabetes increases with worse glycemic control in a particular patient. It is not surprising, therefore, that, as HbA1c increases in diabetic patients, the risk of developing cardiovascular disease (CVD) and the risk of the incidence of cardiovascular events increase [14,15,16,17,18,19]. Therefore, an interesting question can be asked on whether a similar relationship also exists among people who have not been diagnosed with DM. It is worth noting that CVDs are currently one of the most important challenges for public health. Moreover, there is a great need to develop methods to identify patients with subclinical or mildly symptomatic cardiovascular disease and an increased risk of cardiovascular events in order to place these patients under increased surveillance as early as possible and to apply the appropriate diagnostics and treatment at the optimal time [20,21].

The purpose of this paper is to present the results of a narrative review of the literature on the current state of knowledge on the applicability and clinical utility of determining HbA1c in patients without diagnosed DM in the context of assessing the state of the cardiovascular system and the risk of cardiovascular events.

## 2. Glycated Hemoglobin and Subclinical Cardiovascular Dysfunction

### 2.1. The Concept of Subclinical Cardiovascular Dysfunction

The concept of subclinical CVD (or dysfunction) is not precisely defined, but is commonly used in scientific publications in various contexts [22,23,24,25]. In the opinion of the authors of this publication, this term should be understood as features of damage or abnormal function of the cardiovascular system, especially features of endothelial dysfunction, arterial stiffness, and early signs of atherosclerosis development shown in additional tests, without clinical symptoms of overt CVDs, as well as without a history of cardiovascular events. Subclinical atherosclerosis is the presence of atherosclerotic plaques detected in imaging tests without significant stenoses and clinical symptoms of CVDs [26,27]. The tools that can be used to assess subclinical dysfunction of the cardiovascular system include measurement of the intima-media thickness (IMT), especially carotid IMT (cIMT) [28], assessment of endothelial function using the flow-mediated dilation (FMD) method [29], measurement of pulse wave velocity (PWV) [30], the ankle–brachial index (ABI) [31,32], the toe–brachial index (TBI) [33], and the coronary artery calcium (CAC) score [34].

Interestingly, in a study conducted on a group of 219 non-diabetic individuals, significant correlations were found between parameters of the carbohydrate metabolism status, including HbA1c, and the number of individual lymphocyte subpopulations in the peripheral blood, which suggests the existence of a relationship between subclinical fluctuations in the carbohydrate metabolism and the function of the immune system [35]. It should be mentioned that the immune response also plays a key role in the pathogenesis of atherosclerosis [36,37].

### 2.2. Advanced Glycation End Products and Cardiovascular Diseases

In the context of glycated hemoglobin, the concept of advanced glycation end products (AGEs) is worth mentioning. This term refers to a spectrum of molecules that undergo non-enzymatic glycation. The AGE molecule can exert its effect via the receptor of advanced glycation end products (RAGE) [38]. The activity of the AGE–RAGE axis has been discussed in the context of the pathogenesis of numerous diseases, such as cardiovascular diseases [39], diabetic nephropathy [40], and neurodegenerative diseases [41], among others.

The activation of the AGE–RAGE axis via NF-κB contributes to an increased expression of proatherogenic factors such as endothelin-1, intercellular adhesion molecule-1 (ICAM-1), vascular cell adhesion molecule-1 (VCAM-1), E-selectin, tissue factor, tumor necrosis factor (TNF-α), and interleukin (IL-6) [42].

### 2.3. Glycated Hemoglobin and the Presence of Atherosclerotic Plaque in Imaging Diagnosis

Rossello et al. presented the results of a study involving 3973 people aged 40–54 who had not been diagnosed with diabetes, chronic kidney disease, or CVDs. The presence of atherosclerotic lesions was assessed using two-dimensional ultrasonography and computed tomography (CT) without contrast. It was found that the HbA1c value systematically significantly increased the risk of atherosclerotic plaques in the carotid arteries, infrarenal aorta, iliac, and femoral arteries, as did the coronary artery calcium score [43].

Zhu et al. presented the results of a retrospective analysis involving 216 non-diabetic individuals. HbA1c was shown to be significantly higher in patients with carotid atherosclerosis than in patients without carotid atherosclerosis (5.7 ± 0.4% vs. 5.5 ± 0.4%; *p* = 0.009). The authors defined carotid atherosclerosis as the presence of an atherosclerotic plaque or increased cIMT. Notably, the effect of increased HbA1c on the presence of carotid atherosclerosis in non-diabetic patients was even more pronounced when it coexisted with an increased systolic blood pressure value [44].

Lee et al. obtained different conclusions. In a large population-based study (the Dong-gu study) conducted in South Korea, 6500 people without type 2 diabetes aged 50 years or older were included in the final analysis. There was no significant relationship between the HbA1c value, the presence of atherosclerotic plaques, and the luminal diameter [45].

It is worth noting that the studies cited had significant methodological differences. Rossello’s study used imaging data from multiple vascular beds, and the study population was younger than that in the other studies, which assessed only the carotid arteries. There were also significant differences in the cardiovascular status between the study groups. In the study by Zhu et al., 42.6% of patients had a history of coronary artery disease (CAD), whereas in the study by Lee et al., those with a history of CAD were excluded.

### 2.4. Glycated Hemoglobin and Intima-Media Thickness

In a study conducted in China, which included 1627 people aged at least 40 years without carbohydrate metabolism disorders, it was found that the HbA1c value showed a statistically significant positive relationship with the cIMT value, independently of factors such as age, sex, body mass index (BMI), smoking and drinking status, blood pressure, serum lipid levels, and even fasting plasma glucose and post-challenge glucose [46]. The study performed by Alizargar et al. aimed to analyze the factors determining the value of cIMT. The study involved 331 people, 38 of whom were diagnosed with DM. A significant correlation between HbA1c and cIMT was found both among people with DM (r = 0.35, *p* = 0.049), as well as among people without DM (r = 0.196, *p* = 0.002), but among people with DM, the correlation was stronger [47].

Toulis et al. presented the results from The Guangzhou Biobank Cohort Study, Cardiovascular Disease Subcohort, in which 1223 individuals with no history of diabetes participated. Logistic regression analysis showed no significance of HbA1c in the prediction of cIMT and aortic arch calcification in the unadjusted model and in the model adjusted for age and sex. On the other hand, linear regression analysis revealed a significant association between cIMT and HbA1c. It should be noted that, among the whole study population, diabetes was diagnosed in 120 patients (9.8%) based on an oral glucose tolerance test and 138 individuals (11.3%) based on their HbA1c value, so in the results, patients with newly diagnosed DM also participated [48].

However, it should be noted that not all studies suggest a relationship between HbA1c and cIMT. The study conducted by Santos et al. involved 8028 people aged 35–74 without DM and overt CVDs. It was found that, although the HbA1c value was correlated significantly with the homeostasis model assessment—insulin resistance (HOMA-IR) value, no significant relationship was found between cIMT and HbA1c, as well as between cIMT and fasting and post-load glucose serum concentration [49]. Similarly, in the previously cited publication regarding the results of the Dong-gu study, no significant relationship was found between the HbA1c value and the cIMT value [45]. Ziemann et al. presented the results of a study in which the relationship between the HbA1c value and features of cardiovascular dysfunction in older adults without DM was assessed. The study involved 830 people aged 65 or over. There was no significant relationship between the HbA1c and cIMT values in the study population [50].

A relationship between HbA1c and cIMT has been found in patients with prediabetes, which may be characterized by impaired fasting glucose, impaired glucose tolerance, or both. The study, which involved 250 persons, examined the effect of a combined approach of yoga therapy used for six months on cIMT and the metabolic parameters in patients with prediabetes. HbA1c was shown to correlate significantly with cIMT both at the beginning (r = 0.055, *p* = 0.03) and after the intervention (r = 0.514, *p* = 0.01). It should be noted that the correlation was much stronger at the end of the follow-up, when the mean HbA1c value was lower than at the beginning of the study (5.67 ± 0.40% vs. 6.42 ± 0.86%) [51].

### 2.5. Glycated Hemoglobin and Pulse Wave Velocity

In 2018, the results of a study involving 220 non-diabetic people (subsample from the EVIDENT II study) were presented. Statistically significant differences were found between the average PWV values depending on membership in individual tertiles regarding HbA1c values. The values are presented in Table 2. Notably, no significant differences were found when taking into consideration fasting plasma glucose instead of HbA1c [52]. Moreover, in the study conducted by McEniery et al., which involved 4386 people without diabetes, several years of observation showed that the HbA1c value is an independent predictor of the development of arterial stiffness [53]. Similarly, the previously cited study, The Dong-gu study, showed that the HbA1c value was independently associated with the brachial–ankle PWV value in a multivariate linear regression analysis in patients without type 2 diabetes [45]. Another study involving 263 African Americans without diabetes but with insulin resistance showed that, in a fully adjusted multivariate logistic regression analysis, HbA1c predicted higher values of PWV (OR 1.79; 95% CI: 1.09–2.93; *p* = 0.022), as well as higher values of left ventricle mass (OR 1.56; 95% CI: 1.08–2.88; *p* = 0.029) [54].

In a study conducted on a group of 3048 people (Chinese Han population), participants of the Beijing Health Management Cohort (BHMC) study, it was found that HbA1c correlated better than fasting and postprandial blood glucose with arterial stiffness (defined in the cited publication as the presence of an increased brachial–ankle pulse wave velocity or a decreased ankle–brachial index), both in the general population, as well as in the non-diabetic group [55].

Wen et al. conducted a study involving 3640 people, including those with DM (319 people) and those without DM. Arterial stiffness was assessed using ankle–brachial PWV measurement. In the whole study population, HbA1c was correlated significantly with the PWV value (r = 0.355; *p* < 0.001). It was found using logistic regression that, in the multivariate analysis model, the PWV value was significantly predicted by HbA1c both in people with diabetes (OR 1.31; 95% CI 1.06–1.62) and in people without diabetes (OR 1.54; 95% CI 1.09–2.20) [56]. Another study conducted in China on a group of 11,014 people from the general population (14.3% of people had diabetes) also confirmed a significant relationship between the HbA1c value and arterial stiffness assessed using the brachial–ankle PWV value. In particular, it was found that HbA1c predicted arterial stiffness in people without carbohydrate metabolism disorders, both among men (OR 1.39; 95% CI 1.04–1.87) and women (OR 1.46; 95% CI 1.09–1.98) [57]. Similarly, in a study conducted in Korea on a group of 2777 healthy men aged 47.1 ± 9.4 years, it was found that the HbA1c value significantly correlated with the brachial–ankle PWV value (r = 0.144; *p* < 0.001) [58].

In 2023, Saz-Lara et al. presented a proposal for an indicator of cardiovascular risk in the population of healthy people (“early vascular aging”). It was found that a single-factor model, which included parameters such as pulse pressure, HbA1c, pulse wave velocity, and advanced glycation end product concentration, showed the best construct validity for the early vascular aging index [59]. In 2022, Cao et al. presented the results of a study involving 4739 people aged at least 40 years without diabetes or diagnosed CVDs. Interestingly, it was found that prediabetes, diagnosed based on the results of an oral glucose tolerance test and HbA1c value, was associated with subclinical atherosclerosis, understood as increased brachial–ankle PWV and cIMT values, only in the case of people under 60 years of age [60].

In 2013, Shen et al. presented a study in which the relationship between carbohydrate metabolism parameters and arterial stiffness in people with prediabetes was assessed. The study involved 1122 people aged 55 or younger. The fasting plasma glucose and HbA1c were measured in each study participant. It was shown that, in people with HbA1c values of 5.7–6.4% and impaired fasting glucose, the brachial–ankle PWV (1418 ± 27 cm/s) value was higher than that in people with normoglycemia (1282 ± 8 cm/s), in people with HbA1c values of 5.7–6.4% but normal fasting glucose (1311 ± 10 cm/s), as well as in people with impaired fasting glycemia without elevated HbA1c (1398 ± 30 cm/s) (*p* < 0.001 for difference among all groups by ANOVA) [61].

### 2.6. Glycated Hemoglobin and Ankle–Brachial Index

The study conducted by Tanaka et al. involved 4756 people without a history of peripheral arterial disease. The study participants were divided according to their ABI value into a group of people with borderline ABI (0.91–1.00; 324 participants) and a group with normal ABI (1.01–1.39; 4432 participants). It was found that, among people with a borderline ABI value, the average HbA1c value was significantly higher than that among people with a normal ABI value (6.3 ± 1.2 vs. 6.2 ± 1.0; *p* = 0.049). It should be noted, however, that among people with a borderline ABI value, the percentage of people with diabetes was significantly higher than that among people with a normal ABI value (32.1% vs. 23.0%; *p* < 0.001) [62]. Liu et al. 2019 presented the results of a study that assessed the relationship between the HbA1c value and the ABI value. The study involved 3102 subjects aged 67.72 ± 10.69 years. The study was conducted in China as part of the Beijing Vascular Disease Evaluation Study (BEST Study). The study also included people with diabetes, constituting 34.6% of the study population. However, it was shown that, in a multivariate analysis, also taking into account diabetes, the HbA1c value was significantly associated with an increased risk of a reduced ABI value (OR 1.303; 95% CI 1.204–1.410; *p* < 0.001) [63].

However, the research results also showed no significant relationship between the HbA1c value and a reduced ABI value in people without DM. In the previously cited study on the relationship between the HbA1c value and cardiovascular parameters in elderly people without diagnosed DM, no significant relationship was found between HbA1c and the ABI value [50].

## 3. Glycated Hemoglobin and Heart Diseases

The HbA1c value may be a helpful indicator for assessing the risk of CAD in patients without DM, because research shows that this parameter correlates with the presence and severity of changes in coronary arteries in CT examination. In 2019, Ewid et al. presented the results of a study involving 38 non-diabetic patients without diagnosed CAD with a median HbA1c value of 5.7% (range 4.7–6.4%). Coronary arteries were assessed using CT. A moderate correlation was found between the HbA1c value and coronary artery stenosis percentages (r = 0.47, *p* < 0.05). Moreover, HbA1c was shown to be correlated with the number of affected coronary vessels (r = 0.53, *p* < 0.001) [64]. Another study, conducted on 18,504 adult males without DM and CVD, assessed the relationship between the HbA1c value and calcifications in the coronary arteries assessed by CT. It was found that, in subsequent percentiles in terms of HbA1c values, the percentage of patients with calcifications in the coronary arteries increased (9.4%; 11.1%; 14.1%; and 17.3%, respectively; *p* < 0.001) [65].

Similar conclusions result from studies that assessed the relationship between HbA1c and the presence and severity of changes in the coronary arteries, which were assessed using classic invasive coronary angiography. A study involving 299 patients without DM and without a previous history of coronary revascularization showed that the HbA1c value is an independent predictor of the diagnosis of CAD (OR 2.8, 95% CI 1.3–6.2, *p* = 0.009). Interestingly, the predictive value of HbA1c increased when the high-sensitive C-reactive protein (hs-CRP) value was additionally taken into account, because the risk of CAD increases significantly when both the HbA1c and hs-CRP values are in the upper two quartiles (OR 4.183; 95% CI 1.883–9.290, *p* < 0.0001) [66]. Similarly, another study involving 346 non-diabetic patients with CAD confirmed angiographically showed that an increase in the HbA1c value was associated with a significant increase in the severity of CAD and the SYNTAX score [67]. On the other hand, in another study in which 378 non-diabetic patients participated, there was no significant relationship between the SYNTAX score and HbA1c levels in non-diabetics (*p* = 0.885) [68].

HbA1c correlates with certain aspects related to the structure of atherosclerotic plaques in the coronary arteries, which is important in assessing atherosclerotic plaque instability and the risk of acute coronary syndrome. In the study performed by Li et al., both diabetic and non-diabetic patients participated. Even in non-diabetic patients, elevated HbA1c was shown to be significantly associated with a decreased minimal fibrous cap thickness (β = −14.011, *p* = 0.036) and increased lipid index (β = 290.048, *p* = 0.041) and macrophage index (β = 120.029, *p* = 0.048) [69].

According to a study conducted by Ledo Piñeiro et al., no significant correlation was found between pre-diabetes diagnosis, taking into account the HbA1c value and the risk of worse prognosis in patients with atrial fibrillation [70].

## 4. Glycated Hemoglobin and Cerebrovascular Disease

Heo et al. conducted a study involving 639 people diagnosed with stroke. In non-diabetic subjects, there was no significant relationship between the HbA1c value and the presence of cerebrovascular lesions. In patients with diabetes, such a relationship was found only in univariate analysis. After taking into account the age and the presence of hypertension, also in patients with diabetes, the relationship turned out to be insignificant. The presence of cerebrovascular lesions was assessed by magnetic resonance imaging, taking into account both large-artery disease and small-artery disease (such as leukoaraiosis, microbleeds, or old lacunar infarctions) [71].

In another study by Rozanski et al., HbA1c was positively related to the presence of cerebral white matter disease (WMD) features detected by magnetic resonance imaging. In this study, however, patients with and without diabetes were analyzed together. Among the 512 study participants, 120 people (23%) had diabetes. In the entire study population, the median HbA1c value was 5.8% (IQR: 5.4–6.3), which indicates that, among the people included in the analysis, there were people without diabetes, with pre-diabetes, or people diagnosed with diabetes with well-controlled glycemia [72].

Oh et al. presented the results of a study involving 307 non-diabetic male patients from Korea with diagnosed ischemic stroke and 253 control subjects. Although the study included only people without diabetes, the HbA1c value was significantly higher among people with stroke than in the control group (5.8 ± 0.5% vs. 5.5 ± 0.5%, *p* < 0.01). Being in the highest HbA1c quartile was associated with a significantly increased risk of stroke compared to people in the lowest quartile (OR 9.596; 95% CI 3.859–23.863, *p* < 0.01) [73].

The measurement of HbA1c is helpful for assessing stress hyperglycemia in patients with stroke. The stress hyperglycemia ratio (SHR) is the ratio of the glucose concentration in the venous blood plasma to the HbA1c value. The higher the value of the current glucose serum concentration and the lower the HbA1c value, the more likely it is that the hyperglycemia is accidental and related to stress resulting from an acute illness. It was found that the phenomenon of stress hyperglycemia affects the prognosis in patients with cerebral stroke due to large vessel occlusion. The study conducted by Peng et al. involved 542 people, 73.3% of whom had not been diagnosed with diabetes. It was shown that people with an SHR value in the highest tertile were characterized by a significantly lower probability of achieving a favorable functional outcome compared to people with an SHR value in the lowest tertile [74]. Similar conclusions were obtained in the context of the relationship between stress hyperglycemia and the effectiveness of the thrombolytic treatment of stroke. Shen et al. presented the results of a study in which 341 people took part, among which 264 people had not been diagnosed with diabetes (77.4%). The SHR value was found to be an independent predictor of an increased risk of a poor functional outcome after three months in the whole study population (OR 14.639; 95% CI 4.075–52.589; *p* < 0.001) [75].

## 5. Glycated Hemoglobin and Lower Extremity Ischemia

There are minimal data on the relationship between the percentage of glycated hemoglobin, the risk of developing chronic lower limb ischemia, and its severity in patients without diabetes. Studies on this topic were conducted primarily in diabetic patients, and the data on the existence of such a relationship are not fully clear [76,77,78,79].

Chen et al. presented the results of a study involving 3169 people diagnosed with chronic kidney disease. The study included both people with and without diabetes. The percentage of glycated hemoglobin was significantly associated with the risk of developing chronic lower extremities ischemia (HR 1.16; 95% CI 1.05–1.27, *p* = 0.003) after adjustment for these traditional risk factors [80].

## 6. Future Perspectives

Although not fully unambiguous, the results presented in this publication show certain relationships between the HbA1c value and the presence and severity of cardiovascular damage and dysfunction, both at the overt and subclinical levels. It should be emphasized that further studies are needed, preferably those that would assess subclinical cardiovascular dysfunction using different tools simultaneously.

However, these results do not allow us to conclude that HbA1c should be measured in non-diabetic individuals as part of cardiovascular risk assessment in routine clinical practice. It seems, therefore, that the most rational proposal for further research could be to conduct a prospective observational study that would allow us to verify whether the measurement of the HbA1c value in selected subpopulations of non-diabetic patients can lead to a more precise assessment of the risk for the development of overt CVD and cardiovascular event occurrence in comparison to a model based solely on the assessment of classic cardiovascular risk factors.

## 7. Conclusions

The results presented in this literature review show that HbA1c measurement may be helpful not only in patients with DM, but also in non-diabetic patients. HbA1c correlates with some parameters of cardiovascular dysfunction and the risk for the development of overt CVD. It should be emphasized, however, that the features of subclinical cardiovascular dysfunction and then overt CVD result from the interaction of various factors, not only hyperglycemia. It should be mentioned that glycemia fluctuations, which contribute to increases in HbA1c values, can be at the subclinical level and do not allow for the diagnosis of diabetes.

Furthermore, there were significant methodological differences between studies conducted in this area, as some studies included only people without carbohydrate metabolism disorders (taking into account both diabetes and prediabetes). In contrast, other studies included people without diabetes (not analyzing separately people without carbohydrate metabolism disorders and people with prediabetes). Further research is needed to understand better how the HbA1c levels in non-diabetic patients predict the risk of developing CVD and to understand the factors that will allow for better use of these results in routine clinical practice.

The most important findings of the presented literature review are summarized in Table 3.

## Figures and Tables

**Table 1:** The relationship between HbA1c value and mean serum glucose concentration [9].

HbA1c [%]/[mmol/mol]	Mean Serum Glucose Concentration (95% CI) [mg/dL]/[mmol/L]
5.0/31	97 (76–120)/5.4 (4.2–6.7)
6.0/42	126 (100–152)/7.0 (5.5–8.5)
7.0/53	154 (123–185)/8.6 (6.8–10.3)
8.0/64	183 (147–217)/10.2 (8.1–12.1)
9.0/75	212 (170–249)/11.8 (9.4–13.9)
10.0/86	240 (193–282)/13.4 (10.7–15.7)
11.0/97	269 (217–314)/14.9 (12.0–17.5)
12.0/108	298 (240–347)/16.5 (13.3–19.3)

Abbreviations: HbA1c—percentage of glycated hemoglobin; 95% CI—95% confidence interval.

**Table 2:** The relationship between the percentage of glycated hemoglobin (HbA1c), fasting plasma glucose (FPG), and pulse wave velocity (PWV) in a population of non-diabetic patients, according to the study conducted by Cavero-Redondo et al., 2018 [52].

HbA1c [%]	PWV [m/s]	*p*	FPG [mmol/L]	PWV [m/s]	*p*
<5.30	6.88	0.004	<4.44	7.18	0.066
5.30–5.59	7.06		4.44–4.87	7.26	
>5.6	8.16		≥4.88	7.93	

Abbreviations: HbA1c—percentage of glycated hemoglobin; PWV—pulse wave velocity; FPG—fasting plasma glucose; *p*—according to ANOVA in model adjusted for age, sex, triglyceride, HDL cholesterol, LDL cholesterol, and fat mass (%).

**Table 3:** The most important findings of the presented literature review.

As the percentage of glycated hemoglobin increases, the risk of atherosclerotic changes increases in middle-aged non-diabetic patients [43]. This relationship is not clear in older people [44,45].
Studies on the relationship between the percentage of glycated hemoglobin and the carotid intima-media thickness in non-diabetic patients have unclear results [46,47,48,49,50,51].
Studies conducted so far indicate a relationship between the HbA1c value and the severity of arterial stiffness [52,53,54,55,56,57,58,59,60,61].
The available data on the relationship between the percentage of glycated hemoglobin and the ankle–brachial index in non-diabetic patients are minimal. Further studies are needed [50,62,63].
There is a relationship between the percentage of glycated hemoglobin in non-diabetic individuals, the severity of atherosclerosis in the coronary arteries [64,65,66,67], and morphological features indicating the risk of atherosclerotic plaque instability [69]. However, no relationship was found between the percentage of glycated hemoglobin and the SYNTAX score [68].
Prospective observational studies are needed to assess to what extent taking into account the percentage of glycated hemoglobin in non-diabetic patients may help to assess the risk of developing cardiovascular disease and the occurrence of cardiovascular events in non-diabetic patients.

## References

[1] Banday M.Z., Sameer A.S., Nissar S. (2020). Pathophysiology of diabetes: An overview. Avicenna J. Med..

[2] Eid S., Sas K.M., Abcouwer S.F., Feldman E.L., Gardner T.W., Pennathur S., Fort P.E. (2019). New insights into the mechanisms of diabetic complications: Role of lipids and lipid metabolism. Diabetologia.

[3] Jakubiak G.K., Cieślar G., Stanek A. (2022). Nitrotyrosine, nitrated lipoproteins, and cardiovascular dysfunction in patients with type 2 diabetes: What do we know and what remains to be explained?. Antioxidants.

[4] Gougeon R. (2013). Insulin resistance of protein metabolism in type 2 diabetes and impact on dietary needs: A review. Can. J. Diabetes..

[5] Padilla-Martinez F., Wojciechowska G., Szczerbinski L., Kretowski A. (2021). Circulating nucleic acid-based biomarkers of type 2 diabetes. Int. J. Mol. Sci..

[6] Hong X., Hu Y., Yuan Z., Fang Z., Zhang X., Yuan Y., Guo C. (2022). Oxidatively damaged nucleic acid: Linking diabetes and cancer. Antioxid. Redox Signal..

[7] Shin A., Connolly S., Kabytaev K. (2023). Protein glycation in diabetes mellitus. Adv. Clin. Chem..

[8] Stanaway S.E.R.S., Gill G.V. (2000). Protein glycosylation in diabetes mellitus: Biochemical and clinical considerations. Pract. Diab. Int..

[9] Nathan D.M., Kuenen J., Borg R., Zheng H., Schoenfeld D., Heine R.J., A1c-Derived Average Glucose Study Group (2008). Translating the A1C assay into estimated average glucose values. Diabetes Care.

[10] Dal Canto E., Ceriello A., Rydén L., Ferrini M., Hansen T.B., Schnell O., Standl E., Beulens J.W. (2019). Diabetes as a cardiovascular risk factor: An overview of global trends of macro and micro vascular complications. Eur. J. Prev. Cardiol..

[11] Mućka S., Miodońska M., Jakubiak G.K., Starzak M., Cieślar G., Stanek A. (2022). Endothelial function assessment by flow-mediated dilation method: A valuable tool in the evaluation of the cardiovascular system. Int. J. Environ. Res. Public Health..

[12] Jakubiak G.K., Pawlas N., Cieślar G., Stanek A. (2020). Chronic lower extremity ischemia and its association with the frailty syndrome in patients with diabetes. Int. J. Environ. Res. Public Health.

[13] Jakubiak G.K., Pawlas N., Cieślar G., Stanek A. (2021). Pathogenesis and clinical significance of in-stent restenosis in patients with diabetes. Int. J. Environ. Res. Public Health.

[14] Huang E.S., Liu J.Y., Moffet H.H., John P.M., Karter A.J. (2011). Glycemic control, complications, and death in older diabetic patients: The diabetes and aging study. Diabetes Care.

[15] Nichols G.A., Rosales A.G., Perrin N.A., Fortmann S.P. (2014). The association between different A1C-based measures of glycemia and risk of cardiovascular disease hospitalization. Diabetes Care.

[16] Menon V., Kumar A., Patel D.R., John J.S., Wolski K.E., McErlean E., Riesmeyer J.S., Weerakkody G., Ruotolo G., Cremer P.C. (2020). Impact of baseline glycemic control on residual cardiovascular risk in patients with diabetes mellitus and high-risk vascular disease treated with statin therapy. J. Am. Heart Assoc..

[17] Manosroi W., Phimphilai M., Waisayanand N., Buranapin S., Deerochanawong C., Gunaparn S., Phrommintikul A., Wongcharoen W., CORE-Thailand investigators (2023). Glycated hemoglobin variability and the risk of cardiovascular events in patients with prediabetes and type 2 diabetes mellitus: A post-hoc analysis of a prospective and multicenter study. J. Diabetes Investig..

[18] Li Q., Yuan D., Zeng G., Jiang L., Xu L., Xu J., Liu R., Song Y., Zhao X., Hui R. (2024). The association between glycated hemoglobin levels and long-term prognosis in patients with diabetes and triple-vessel coronary disease across different age groups: A cohort study. Diabetes Res. Clin. Pract..

[19] Tang X.F., Li Q.X., Han Y.L., Wang X.Z., Song Y., Zhang Z., Xu J.J., Liu Z.Y., Chen Y., Zhang Y.Z. (2024). Implications of baseline glycemic control by plasma glycated hemoglobin A1c on adverse outcomes in patients with coronary heart disease and type 2 diabetes mellitus: Results from the PROMISE study. Heliyon.

[20] Starzak M., Stanek A., Jakubiak G.K., Cholewka A., Cieślar G. (2022). Arterial stiffness assessment by pulse wave velocity in patients with metabolic syndrome and its components: Is it a useful tool in clinical practice?. Int. J. Environ. Res. Public Health.

[21] Jakubiak G.K. (2024). Cardiac troponin serum concentration measurement is useful not only in the diagnosis of acute cardiovascular events. J. Pers. Med..

[22] Gomez J.M.D., VanHise K., Stachenfeld N., Chan J.L., Merz N.B., Shufelt C. (2022). Subclinical cardiovascular disease and polycystic ovary syndrome. Fertil. Steril..

[23] Miner M., Parish S.J., Billups K.L., Paulos M., Sigman M., Blaha M.J. (2019). Erectile dysfunction and subclinical cardiovascular disease. Sex. Med. Rev..

[24] Fang M., Wang D., Tang O., McEvoy J.W., Echouffo-Tcheugui J.B., Christenson R.H., Selvin E. (2023). Subclinical cardiovascular disease in US adults with and without diabetes. J. Am. Heart Assoc..

[25] Blachut D., Przywara-Chowaniec B., Mazurkiewicz M., Tomasik A. (2024). Assessment of arterial stiffness and biochemical markers in systemic lupus erythematosus in the diagnosis of subclinical atherosclerosis. J. Pers. Med..

[26] Soni M., Ambrosino M., Jacoby D.S. (2021). The use of subclinical atherosclerosis imaging to guide preventive cardiology management. Curr. Cardiol. Rep..

[27] Fernández-Friera L., Ibáñez B., Fuster V. (2014). Imaging subclinical atherosclerosis: Is it ready for prime time? A review. J. Cardiovasc. Transl. Res..

[28] Bengtsson A., Nyman E., Grönlund C., Wester P., Näslund U., Fhärm E., Norberg M. (2023). Multi-view carotid ultrasound is stronger associated with cardiovascular risk factors than presence of plaque or single carotid intima media thickness measurements in subclinical atherosclerosis. Int.J. Cardiovasc. Imaging.

[29] Thijssen D.H.J., Bruno R.M., van Mil A.C.C.M., Holder S.M., Faita F., Greyling A., Zock P.L., Taddei S., Deanfield J.E., Luscher T. (2019). Expert consensus and evidence-based recommendations for the assessment of flow-mediated dilation in humans. Eur. Heart J..

[30] Milan A., Zocaro G., Leone D., Tosello F., Buraioli I., Schiavone D., Veglio F. (2019). Current assessment of pulse wave velocity: Comprehensive review of validation studies. J. Hypertens..

[31] Yang Y., Liu L., Sun H., Nie F., Hu X. (2020). Relation between high ankle-brachial index and cardiovascular outcomes in the general population and cardiovascular disease: A meta-analysis. Int. Angiol..

[32] Cáceres-Farfán L., Moreno-Loaiza M., Cubas W.S. (2021). Ankle-brachial index: More than a diagnostic test?. Arch. Peru Cardiol. Cir. Cardiovasc..

[33] Darban Hosseini Amirkhiz G., Babaei M.R., Madani N.H., Khamseh M.E. (2021). Toe-brachial index is beyond a peripheral issue in patients with type 2 diabetes. PLoS ONE.

[34] Hussain B., Mahmood A., Flynn M.G., Alexander T. (2023). Coronary artery calcium scoring in asymptomatic patients. HCA Healthc. J. Med..

[35] Schmitz T., Freuer D., Linseisen J., Meisinger C. (2024). Associations between blood markers of glucose metabolism and characteristics of circulating lymphocytes. Clin. Nutr..

[36] Good E., Åkerman L., Nyström S., Jonasson L., Ernerudh J., de Muinck E. (2023). Changes in natural killer and T lymphocyte phenotypes in response to cardiovascular risk management. Sci. Rep..

[37] Bashore A.C., Yan H., Xue C., Zhu L.Y., Kim E., Mawson T., Coronel J., Chung A., Ho S., Ross L.S. (2024). High-dimensional single-cell multimodal landscape of human carotid atherosclerosis. Arterioscler. Thromb. Vasc. Biol..

[38] Wang B., Jiang T., Qi Y., Luo S., Xia Y., Lang B., Zhang B., Zheng S. (2024). AGE-RAGE axis and cardiovascular diseases: Pathophysiologic mechanisms and prospects for clinical applications. Cardiovasc. Drugs Ther..

[39] Prasad K. (2021). AGE-RAGE stress and coronary artery disease. Int. J. Angiol..

[40] Sanajou D., Ghorbani Haghjo A., Argani H., Aslani S. (2018). AGE-RAGE axis blockade in diabetic nephropathy: Current status and future directions. Eur. J. Pharmacol..

[41] Raghavan C.T. (2024). Advanced glycation end products in neurodegenerative diseases. J. Mol. Neurosci..

[42] Singh S., Siva B.V., Ravichandiran V. (2022). Advanced glycation end products: Key player of the pathogenesis of atherosclerosis. Glycoconj. J..

[43] Rossello X., Raposeiras-Roubin S., Oliva B., Sánchez-Cabo F., García-Ruíz J.M., Caimari F., Mendiguren J.M., Lara-Pezzi E., Bueno H., Fernández-Friera L. (2021). Glycated hemoglobin and subclinical atherosclerosis in people without diabetes. J. Am. Coll. Cardiol..

[44] Zhu W., Sun T., Shi H., Li J., Zhu J., Qi W., Luo X., Li Y. (2010). Combined effects of glycated hemoglobin A1c and blood pressure on carotid artery atherosclerosis in nondiabetic patients. Clin. Cardiol..

[45] Lee Y.H., Shin M.H., Choi J.S., Rhee J.A., Nam H.S., Jeong S.K., Park K.S., Ryu S.Y., Choi S.W., Kim B.H. (2016). HbA1c is significantly associated with arterial stiffness but not with carotid atherosclerosis in a community-based population without type 2 diabetes: The Dong-gu study. Atherosclerosis.

[46] Huang Y., Bi Y., Wang W., Xu M., Xu Y., Li M., Wang T., Chen Y., Li X., Ning G. (2011). Glycated hemoglobin A1c, fasting plasma glucose, and two-hour postchallenge plasma glucose levels in relation to carotid intima-media thickness in Chinese with normal glucose tolerance. J. Clin. Endocrinol. Metab..

[47] Alizargar J., Bai C.H. (2018). Factors associated with carotid Intima media thickness and carotid plaque score in community-dwelling and non-diabetic individuals. BMC Cardiovasc. Disord..

[48] Toulis K.A., Jiang C.Q., Hemming K., Nirantharakumar K., Cheng K.K., Lam T.H., Thomas G.N. (2018). Glycated hemoglobin, albuminuria and surrogate markers of macrovascular disease in adults without diabetes: The Guangzhou Biobank Cohort Study, Cardiovascular Disease Subcohort. Can. J. Diabetes.

[49] Santos I.S., Bittencourt M.S., Goulart A.C., Schmidt M.I., Diniz M.F.H.S., Lotufo P.A., Benseñor I.M. (2017). Insulin resistance is associated with carotid intima-media thickness in non-diabetic subjects. A cross-sectional analysis of the ELSA-Brasil cohort baseline. Atherosclerosis.

[50] Zieman S.J., Kamineni A., Ix J.H., Barzilay J., Djoussé L., Kizer J.R., Biggs M.L., de Boer I.H., Chonchol M., Gottdiener J.S. (2012). Hemoglobin A1c and arterial and ventricular stiffness in older adults. PLoS ONE.

[51] Saboo N., Kacker S. (2023). A study to assess and correlate metabolic parameters with carotid intima-media thickness after combined approach of yoga therapy among prediabetics. Adv. Biomed. Res..

[52] Cavero-Redondo I., Martínez-Vizcaíno V., Álvarez-Bueno C., Recio-Rodríguez J.I., Gómez-Marcos M.Á., García-Ortiz L. (2018). Relationship between glycaemic levels and arterial stiffness in non-diabetic adults. Med. Clin..

[53] McEniery C.M., Wilkinson I.B., Johansen N.B., Witte D.R., Singh-Manoux A., Kivimaki M., Tabak A.G., Brunner E.J., Shipley M.J. (2017). Nondiabetic glucometabolic status and progression of aortic stiffness: The Whitehall II study. Diabetes Care.

[54] Stakos D.A., Schuster D.P., Sparks E.A., Meis S.B., Wooley C.F., Osei K., Boudoulas H. (2007). Association between glycosylated hemoglobin, left ventricular mass and aortic function in nondiabetic individuals with insulin resistance. Eur. J. Endocrinol..

[55] Han Z., Kang X., Zhang J., Wang J., Liu Y., Liu J., Wu Z., Li X., Zhao X., Guo X. (2022). Glycated hemoglobin and risk of arterial stiffness in a Chinese Han population: A longitudinal study. Front. Endocrinol..

[56] Wen J., Hu F., Yang Q. (2020). Comparison of hemoglobin Alc, glycated albumin and fasting plasma glucose for prediction of arterial stiffness in Chinese adults. Diabetes Metab. Syndr. Obes..

[57] Zeng Q., Dong S.Y., Wang M.L., Wang W.M., Li J.M., Dai Z.X., Li J., Yang S.W., Zhu L. (2017). Serum glycated albumin, glycated hemoglobin, and arterial stiffness in a general Chinese population. Clin. Chim. Acta.

[58] Noh J.W., Kim E.J., Seo H.J., Kim S.G. (2016). Independent association between glycated hemoglobin and arterial stiffness in healthy men. J. Diabetes Investig..

[59] Saz-Lara A., Cavero-Redondo I., Pascual-Morena C., Martínez-García I., Rodríguez-Gutiérrez E., Lucerón-Lucas-Torres M., Bizzozero-Peroni B., Moreno-Herráiz N., Martínez-Rodrigo A. (2023). Early vascular aging as an index of cardiovascular risk in healthy adults: Confirmatory factor analysis from the EVasCu study. Cardiovasc. Diabetol..

[60] Cao Q., Xin Z., He R., Wang T., Xu M., Lu J., Dai M., Zhang D., Chen Y., Zhao Z. (2022). Age-specific difference in the association between prediabetes and subclinical atherosclerosis: An analysis of a Chinese prospective cohort study. Cardiovasc. Diabetol..

[61] Shen L., Zhang Y.G., Liu M., Qiang D.C., Sun X.L., Liu L., Jiang Y.Y. (2013). Increased arterial stiffness in subjects with pre-diabetes among middle aged population in Beijing, China. Biomed. Environ. Sci..

[62] Tanaka S., Kaneko H., Kano H., Matsuno S., Suzuki S., Takai H., Otsuka T., Uejima T., Oikawa Y., Nagashima K. (2016). The predictive value of the borderline ankle-brachial index for long-term clinical outcomes: An observational cohort study. Atherosclerosis.

[63] Liu H., Liu J., Zhao H., Wang H., BEST Research Group (2019). Relationship between glycated hemoglobin and low ankle-brachial index: A cross-sectional observational study from the Beijing Vascular Disease Evaluation Study (BEST Study). Int. Angiol..

[64] Ewid M., Sherif H., Billah S.M.B., Saquib N., AlEnazy W., Ragab O., Enabi S., Rajab T., Awad Z., Abazid R. (2019). Glycated hemoglobin predicts coronary artery disease in non-diabetic adults. BMC Cardiovasc. Disord..

[65] Jung C.H., Rhee E.J., Kim K.J., Kim B.Y., Park S.E., Chang Y., Ryu S., Park C.Y., Mok J.O., Oh K.W. (2015). Relationship of glycated hemoglobin A1c, coronary artery calcification and insulin resistance in males without diabetes. Arch. Med. Res..

[66] Ashraf H., Boroumand M.A., Amirzadegan A., Talesh S.A., Davoodi G. (2013). Hemoglobin A1C in non-diabetic patients: An independent predictor of coronary artery disease and its severity. Diabetes Res. Clin. Pract..

[67] Dutta B., Neginhal M., Iqbal F. (2016). Glycated hemoglobin (HbA1c) correlation with severity of coronary artery disease in non-diabetic patients—A hospital based study from North-Eastern India. J. Clin. Diagn. Res..

[68] Ul-Haque I., Ud Deen Z., Shafique S., Ur Rehman S.I., Zaman M., Basalat S.T., Munaf M., Wahidi Y. (2019). The role of glycated hemoglobin A1c in determining the severity of coronary artery disease in diabetic and non-diabetic subjects in Karachi. Cureus.

[69] Li D., Li Y., Wang C., Jiang H., Zhao L., Hong X., Lin M., Luan Y., Shen X., Chen Z. (2022). Elevation of hemoglobin A1c increases the atherosclerotic plaque vulnerability and the visit-to-visit variability of lipid profiles in patients who underwent elective percutaneous coronary intervention. Front. Cardiovasc. Med..

[70] Ledo Piñeiro A., Abu-Assi E., González Bermúdez I., Noriega Caro V., Íñiguez-Romo A., Raposeiras-Roubín S. (2024). Is pre-diabetes a predictor of events in patients with atrial fibrillation?. Int. J. Cardiol..

[71] Heo S.H., Lee S.H., Kim B.J., Kang B.S., Yoon B.W. (2010). Does glycated hemoglobin have clinical significance in ischemic stroke patients?. Clin. Neurol. Neurosurg..

[72] Rozanski M., Richter T.B., Grittner U., Endres M., Fiebach J.B., Jungehulsing G.J. (2014). Elevated levels of hemoglobin A1c are associated with cerebral white matter disease in patients with stroke. Stroke.

[73] Oh H.G., Rhee E.J., Kim T.W., Lee K.B., Park J.H., Yang K.I., Jeong D., Park H.K. (2011). Higher glycated hemoglobin level is associated with increased risk for ischemic stroke in non-diabetic Korean male adults. Diabetes Metab. J..

[74] Peng Z., Song J., Li L., Guo C., Yang J., Kong W., Huang J., Hu J., Liu S., Tian Y. (2023). Association between stress hyperglycemia and outcomes in patients with acute ischemic stroke due to large vessel occlusion. CNS Neurosci. Ther..

[75] Shen C.L., Xia N.G., Wang H., Zhang W.L. (2022). Association of stress hyperglycemia ratio with acute ischemic stroke outcomes post-thrombolysis. Front. Neurol..

[76] Rhee J.J., Zheng Y., Montez-Rath M.E., Chang T.I., Winkelmayer W.C. (2017). Associations of glycemic control with cardiovascular outcomes among US hemodialysis patients with diabetes mellitus. J. Am. Heart Assoc..

[77] Shatnawi N.J., Al-Zoubi N.A., Hawamdeh H.M., Khader Y.S., Heis M., Al Omari M., Bataineh B. (2021). The relation of anatomical distribution of symptomatic peripheral arterial disease (PAD) with HbA1c level in patients with type 2 diabetes mellitus. Ther. Adv. Endocrinol. Metab..

[78] Hu Y., Ling T., Yu M., Bai Y., Feng T., Zhang P., Wang Y. (2020). Apolipoprotein E gene polymorphism, glycated hemoglobin, and peripheral arterial disease risk in Chinese type 2 diabetic patients. Dis. Markers..

[79] Kim K.J., Choi J., Bae J.H., Kim K.J., Yoo H.J., Seo J.A., Kim N.H., Choi K.M., Baik S.H., Kim S.G. (2021). Time to reach target glycosylated hemoglobin is associated with long-term durable glycemic control and risk of diabetic complications in patients with newly diagnosed type 2 diabetes mellitus: A 6-year observational study. Diabetes Metab. J..

[80] Chen J., Mohler E.R., Xie D., Shlipak M., Townsend R.R., Appel L.J., Ojo A., Schreiber M., Nessel L., Zhang X. (2016). Traditional and non-traditional risk factors for incident peripheral arterial disease among patients with chronic kidney disease. Nephrol. Dial. Transplant..

